# A Hydrophobic Gold Surface Triggers Misfolding and Aggregation of the Amyloidogenic Josephin Domain in Monomeric Form, While Leaving the Oligomers Unaffected

**DOI:** 10.1371/journal.pone.0058794

**Published:** 2013-03-19

**Authors:** Alessandra Apicella, Monica Soncini, Marco Agostino Deriu, Antonino Natalello, Marcella Bonanomi, David Dellasega, Paolo Tortora, Maria Elena Regonesi, Carlo Spartaco Casari

**Affiliations:** 1 Energy Department and NEMAS – Center for NanoEngineered Materials and Surfaces, Politecnico di Milano, Milan, Italy; 2 École Polytechnique Fédérale de Lausanne (EPFL) Département Science et Génie des Matériaux Laboratoire de technologie des composites et polymères (LTC), Lausanne, Switzerland; 3 Dipartimento di Elettronica, Informazione e Bioingegneria, Politecnico di Milano, Milan, Italy; 4 Department of Aerospace and Mechanical Engineering (DIMEAS), Politecnico di Torino, Turin, Italy; 5 Department of Biotechnologies and Biosciences, University of Milano-Bicocca, Milan, Italy; 6 Department of Statistics and Quantitative Methods (DiSMeQ), University of Milano-Bicocca, Milan, Italy; 7 Center for Nano Science and Technology @Polimi, Italian Institute of Technology, Milan, Italy; Universita' di Padova, Italy

## Abstract

Protein misfolding and aggregation in intracellular and extracellular spaces is regarded as a main marker of the presence of degenerative disorders such as amyloidoses. To elucidate the mechanisms of protein misfolding, the interaction of proteins with inorganic surfaces is of particular relevance, since surfaces displaying different wettability properties may represent model systems of the cell membrane. Here, we unveil the role of surface hydrophobicity/hydrophilicity in the misfolding of the Josephin domain (JD), a globular-shaped domain of ataxin-3, the protein responsible for the spinocerebellar ataxia type 3. By means of a combined experimental and theoretical approach based on atomic force microscopy, Fourier transform infrared spectroscopy and molecular dynamics simulations, we reveal changes in JD morphology and secondary structure elicited by the interaction with the hydrophobic gold substrate, but not by the hydrophilic mica. Our results demonstrate that the interaction with the gold surface triggers misfolding of the JD when it is in native-like configuration, while no structural modification is observed after the protein has undergone oligomerization. This raises the possibility that biological membranes would be unable to affect amyloid oligomeric structures and toxicity.

## Introduction

Protein aggregation is nowadays a topic of great and critical interest in protein science, since the hallmark of many degenerative disorders is the accumulation of intra/extracellular amyloid fibrillar aggregates [1]. Although changes in pH, temperature, protein concentration [2] and medium composition [3] can substantially affect protein misfolding and aggregate formation in solution, the adsorption of proteins onto a surface has been also implicated in the process [4]. Indeed, it has been observed that, depending on surface properties, protein deposition on a substrate may result in misfolding and aggregation via several pathways [5]. In particular, hydrophobic substrates favor protein adsorption without the need of additional energy and drive aggregation, as shown by *in vitro* [6] and *in vivo* experiments [7], [8].

In neurodegenerative disorders, cell morphology and membrane permeability seem to be directly affected by the interactions of cellular components with the earliest protein aggregation products, referred to as oligomers. These are small and generally cytotoxic aggregates that, as a rule, arise form misfolded protein [8]. It has been reported that prefibrillar aggregates can interact with both synthetic phospholipid bilayers [9], [10] and cell membranes with resulting loss of membrane integrity, derangement of selective ion permeability, and impairment of specific membrane-bound protein function [11]. For this reason, besides the study of the role of a contact surface on aggregation kinetics of proteins deposited in their native-like state, further investigations on the interaction mechanisms between oligomeric species and surfaces are required. In this perspective, a goal of paramount biological importance is the understanding of the mechanisms by which specific surface features mimicking a biological substrate affect protein misfolding and aggregation. Hydrophilic and hydrophobic substrates are generally accepted as representative models of cell membrane external surface and of the interior of the lipid bilayer, respectively [1]. Many studies have been reported on amyloid proteins adsorbed on inorganic substrates. For example, α-synuclein and Alzheimer's β-amyloid peptides have been investigated in contact with anionic micelles and mica, respectively [4], [12] but no similar studies on natively structured, amyloidogenic proteins have been so far reported. Here, we present investigations aimed at providing insight into the effects of surface properties on the Josephin domain (JD) of ataxin-3 (AT-3), the protein responsible for the spinocerebellar ataxia type 3. AT-3 consists of the N-terminal globular JD followed by a flexible region containing a polyglutamine (polyQ) tract close to the C-terminus, which triggers the neurodegenerative disease when is expanded beyond a critical threshold. It has been shown that in the AT-3 aggregation pathway the first step is reversible, polyQ independent, and proceeds via JD aggregation [13]–[15]. This fits with the observation that the JD in isolation is also capable of giving rise to oligomers under physiological conditions [16], [17], suggestive of an intrinsic amyloidogenic potential. In contrast, the second step is irreversible and polyQ dependent.

We have investigated the effects of two model surfaces, mica and gold (which are hydrophilic and hydrophobic, respectively) on JD aggregation state and conformational properties, before and after a 48-h *in vitro* incubation under physiological conditions. Based on the above remarks, JD aggregation corresponds to the first step of the overall full-length AT-3 fibrillogenesis. It represents therefore a critical event, also involved in the formation of the oligomeric, toxic species.

In these investigations, we have taken advantage of different experimental and theoretical techniques: i) atomic force microscopy (AFM) [18], which makes it possible to detect - with high spatial resolution and without sample damage - the morphology adopted by a protein lying on a surface [19]; ii) Fourier transform infrared (FTIR) spectroscopy, which provides information on secondary structure and aggregation state of proteins in solution and on solid surfaces [6], [17], [20] iii) molecular dynamics (MD) simulations. Computational approaches like molecular modeling have often proven to be helpful in adding valuable quantitative information to experimental data [21]–[23] and in particular MD allows to investigate (with atomic resolution) the dynamics of protein conformational changes as a result of interactions with a specific substrate [2], [24]–[26].

Our results show that hydrophobic substrates exert a different influence on JD structure in its native-like monomeric state compared to the JD oligomers. As major conformational changes are induced by hydrophobic surfaces on monomeric JD only, this suggests that the surface properties cannot modify the aggregate conformation when the JD is assembled into oligomers, nor can they modify their cytotoxic properties against cell membranes.

## Results

### Morphology of proteins adsorbed on mica and gold

**Figure 1:**
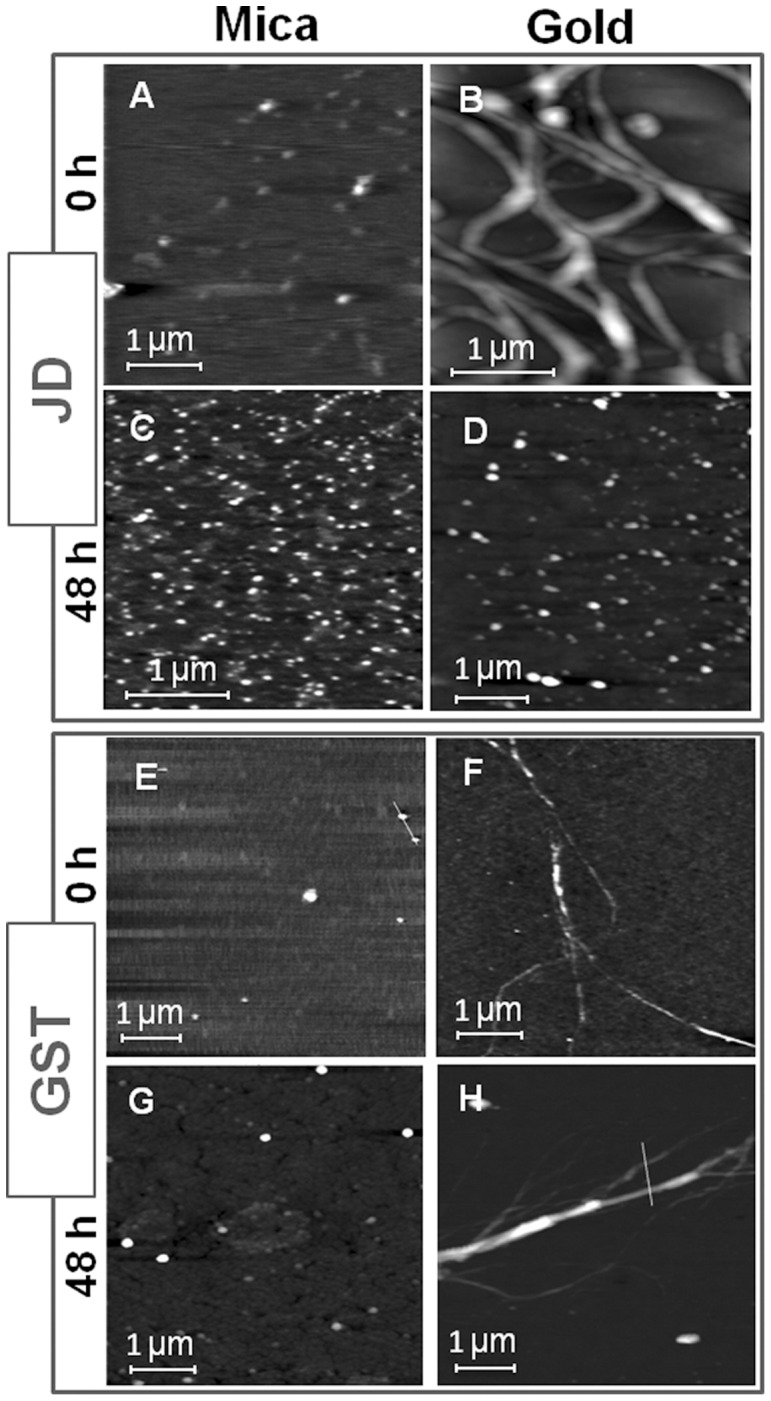
AFM images at t_0_ and t_48_ incubation time of JD on mica (A,C) and gold (B,D) and of GST on mica (E,G) and gold (F,H).

The morphology of JD deposited and dried on both mica and gold, was investigated by AFM, as shown in Figure 1, before and after incubation under physiological conditions. Preliminary circular dichroism (CD) and SEC-FPLC showed that the protein was properly folded and predominantly monomeric (Fig. S1). Both freshly prepared (t_0_) and 48-h incubated (t_48_) JD samples deposited on mica (Fig. 1A and 1C) displayed globular structures. In contrast, on gold, t_0_ samples mostly appeared as bundles of filaments of different dimensions (Fig. 1B), while after incubation JD generated globular structures similar to those observed on mica under the same conditions (Fig. 1D). Such globular structures were prevalently imaged as isolated entities randomly scattered. On mica, at t_0_ and t_48_ their height was about 2±0.5 nm, as measured by AFM. Using deconvolution procedures [19], lateral dimensions between 5 and 25 nm were estimated, irrespective of substrate and incubation time. The smallest size fits with that expected for a single JD molecule [27], suggestive of a monomeric structure, while larger sizes are representative of oligomers consisting of few monomers. From AFM analysis only, we were not able to discriminate between monomeric or oligomeric structures. Elongated structures in the form of filaments packed in bundles were mainly observed in freshly prepared samples deposited on gold (Fig. 1B). Such filaments, with a height distribution in the 5–25 nm range and lateral size of about 100–200 nm, are characterized by a high number of bent regions indicating a highly flexible structure. AFM observations were confirmed by scanning electron microscopy (SEM) images taken on wider sample areas (Fig. S2). Substrates can mediate filaments formation due to adsorption phenomena also related to drop drying [28], this effect being stronger on hydrophobic materials than on hydrophilic ones (Fig. S3).

To further assess the effect of surface properties on protein aggregation, for comparison we also investigated how the deposition on both substrates affects GST aggregation state, a well-known globular and non-amyloidogenic protein, similar in size to the JD [29]. Due to these properties, under physiological conditions GST does not exhibit any tendency to self-assemble in solution, unlike JD. Although only monomeric GST was observed on mica (Fig. 1E and 1G), filaments were imaged on gold at both t_0_ and t_48_ (Fig. 1F and 1H). On the whole, these results confirm that interactions with the hydrophobic substrate can trigger aggregation of the amyloidogenic JD in its native conformation only, an event that is most likely mediated by misfolding. In contrast, this substrate is apparently unable to affect the conformation of oligomeric aggregates arising from protein incubation. This is paralleled by extensive denaturation both before and after incubation. However, as detected by FTIR spectroscopy, only after incubation did the protein generate intermolecular β-sheets, which are the hallmark of amyloid aggregation, (see next paragraph).

### FTIR analysis of proteins in solution and adsorbed on surfaces

**Figure 2:**
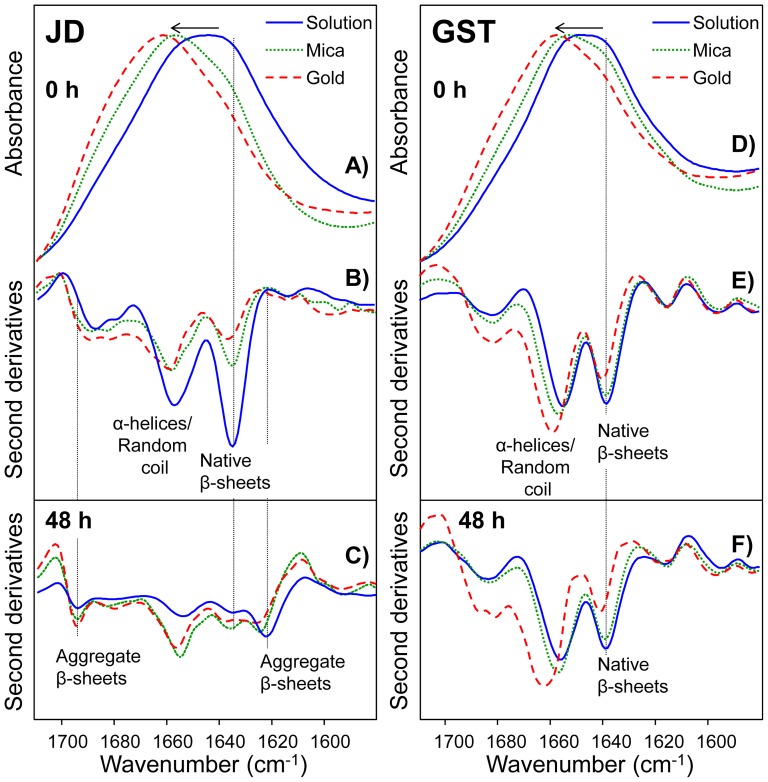
Secondary structures of JD and GST as detected by FTIR spectroscopy. FTIR absorption spectra, in attenuated total reflection mode (ATR), of JD at t_0_ h in solution, on mica and on gold are shown in the Amide I region (A). Second derivative spectra of JD at t_0_ (B) and at t_48_ (C) in solution, on mica and on gold are reported after normalization at the ∼1515 cm^−1^ Tyr peak. FTIR/ATR absorption spectra of GST at t_0_ in solution, on mica and on gold are shown in the Amide I region (D). Second derivative spectra of GST at t_0_ (E) and at t_48_ (F) in solution, on mica and on gold are reported after normalization at the ∼1515 cm-1 Tyr peak. The band assignment of the main peaks to the protein secondary structures are indicated.

The effect of surface properties on JD and GST secondary structure was investigated by FTIR spectroscopy, as shown in Figure 2, where protein absorption spectra, and their second derivatives, are reported at t_0_ and t_48_. After this time, no further changes in FTIR spectra were detected under our working conditions (Fig. S4A). In parallel, we also monitored aggregation kinetics by ThT fluorescence, which leveled off after about 10 h of incubation (Fig. S4B). In the case of JD, a shift of the Amide I band (1600–1700 cm^−1^ spectral range) maximum toward higher wavenumbers was observed at t_0_ when going from JD in solution to JD in contact with mica or gold substrates (see arrow in Fig. 2A), which indicates an unfolding of native α-helices and β-sheets structures. This shift also results in major changes in the second derivative spectra (Fig. 2B), where the component at 1657 cm^−1^ (assigned to α-helices and random coils) and the one at 1635 cm^−1^ (due to β-sheets) [17] decreased in intensity when proteins were adsorbed on surfaces. The unfolding of the native secondary structures is more evident on gold than on mica (Fig. 2A and 2B). Second derivatives spectra of the insoluble JD aggregates at t_48_ in solution and on surfaces (Fig. 2C) displayed in all cases intermolecular β-sheets in their structure, which instead were not detected in the t_0_ samples. Therefore, after a 48-h incubation in solution, the contact between aggregated JD and either hydrophilic or hydrophobic surfaces exerts only a minor effect on oligomer secondary structures. Mass spectra of JD under denaturing conditions determined before and after a 48-h incubation (Fig. S5) show that the oxidation state of the protein was not appreciably changed after incubation, providing further evidence that protein aggregation occurs only as a result of conformational rearrangements. Remarkably, under the same conditions, changes in GST native secondary structure induced by interaction with either substrates at both t_0_ and t_48_, were much smaller than those observed for JD at t_0_. However, these effects were more evident in the case of GST-gold interaction (Fig. 2D–F). In particular, the ∼1638 cm^−1^ component (assigned to native β-sheets) decreased in intensity and the ∼1655 cm^−1^ upshifted to ∼1659 cm^−1^ also displaying higher intensity. Since the absorption of both α-helices and disordered structures occurs in this spectral region, these changes suggest a partial unfolding and/or rearrangement of the native α-helices. Thus, FTIR spectra indicate that at both t_0_ and t_48_ the gold substrate induces minor structural changes on GST compared to those observed for the protein in solution, whereas in contrast, at t_0_ JD interaction with gold surfaces results in a substantially different behavior compared to the protein in solution. On the whole, our results show that the interaction of the amyloidogenic JD with the hydrophobic substrate causes misfolding and aggregation only provided no oligomerization has taken place. They also show that the resulting aggregates at t_0_ are quite likely amorphous, as clearly supported by the absence of intermolecular β-sheets. In contrast, the aggregation and conformational state of the non-amyloidogenic GST is affected by the substrate properties irrespective of the incubation time.

### Protein conformational analysis by MD simulations

**Figure 3:**
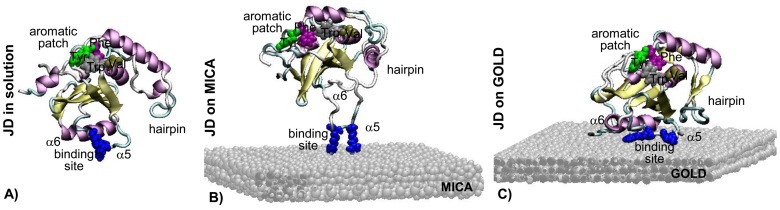
Structure of JD in physiological-like solvated environment (A) in contact with hydrophilic mica surface (B) and hydrophobic gold surface (C). Gold and mica are represented in grey. JD secondary structure is visualized by arrows for β-sheet structures, helices for α-helix structures and tubes for coils and turns. Specific residues are enlightened with VDW representation: the surface binding site, consisting of Arg 101 and Arg 103 residues, and the solvent-exposed aromatic patch forming a binding site specific for ubiquitin-like proteins consisting of Val86 and Trp87 spatially close to Tyr27 and Phe27.

To provide a deeper insight into substrate-induced structural changes of JD, we also performed conformational analyses by MD simulations in a physiological-like solvated environment. They revealed that, in the presence of hydrophilic or hydrophobic surfaces, the spatial conformation and the secondary structures of the protein are altered. An interesting outcome is that the JD protein approaches and binds the surface with the same binding site, irrespective of the initial orientation with respect to the surface, and of the nature of the surface (Fig. 3). The identified binding site consists of Arg101 and Arg103 residues of the α5-helix. However, our results suggest that the mode of interaction of the binding site is substantially different depending on the substrate, the two residues binding to gold via their aliphatic moieties; with mica via their charged guanidino groups. This also results in major differences in the contact surface between JD protein and the substrates, and in protein conformation, as outlined below.

The contact surface was calculated as half of the difference between the surface available to the solvent (SAS) of JD and substrate in isolation, and the SAS of JD/substrate molecular complex. The JD in contact with gold showed a 9-nm^2^ wide contact surface, involving mainly the region containing residues Arg101 and Arg103 and the α6-helix (Fig. S6A and S6B). Most of the contact area between JD and gold (7.5 nm^2^) is hydrophobic. In contrast, the JD-mica system revealed a very low contact surface (0.75 nm^2^, Fig. S6C) due to the peculiar JD conformation on mica: indeed, the protein interacts via Arg101 and Arg103 residues only (Fig. S6D). To better investigate the role of the Arg101 and Arg103 residues in the binding process, Ala scan MD simulations (100 ns) were performed with residues in position 101 and 103 being replaced by Ala. Ala scan MD simulations revealed a reduction in the contact surface when JD interacts with gold (from 9 nm^2^ to 6.5 nm^2^, Fig. S6A): the contact with residues in position 101 and 103 is lost and JD interacts with the surface via the α3-helix of the hairpin. In the case of JD-mica molecular system, the Ala scan simulation showed the detachment of JD from mica surface (the contact surface became zero after 3 ns of MD simulation, Fig. S6C). The modification of residues in position 101 and 103 implies the detachment of JD from the mica surface, revealing a key role of these two residues for the JD binding process.

The JD in solvated environment (Fig. 3A) has a constant radius of gyration (Rg ≈ 1.74 nm during the last 50 ns of MD simulation), a slightly close and fluctuating α2–α3 hairpin (Fig. S7), a well-conserved secondary structure, and a well-defined and compact ubiquitin-binding site. Here, residues Val86 and Trp87 are close to Tyr27 and Phe28, forming a solvent-exposed aromatic patch, recognized as one of the most highly aggregation-prone in JD [30]. When JD is faced to the mica surface (Fig. 3B), the protein largely retains its native structure. Still, Arg101 and Arg103 residues are in close contact with the mica surface, while the rest of the protein is positioned far from the surface (around 2 nm). Arg 101 and Arg 103 represent the unique binding site between JD and mica substrate, thus inducing the JD peculiar conformation where α5-helix is highly stretched and completely unfolded. After 50 ns of MD simulation, the α2–α3 hairpin suddenly moves close to the protein globular domain, achieving a stably closed conformation (Fig. S7), Rg decreases from 1.85 nm to about 1.65 nm, maintained from 50 to 500 ns. In this conformation, the β-core domain of the JD is completely hidden by the hairpin in the back region. A peculiar aspect observed for the JD-mica system is a major change in the conformation of the aromatic patch. Indeed, the two hydrophobic sites (Tyr27–Phe28 and Val86–Trp87) are spaced apart and arranged in a linear sequence on JD external surface. We argue that this conformation of the aromatic site could reduce the aggregation propensity, with resulting formation of globular structures only, as observed in AFM experiments. Unlike the mode of interaction with the mica substrate, in the presence of gold (Fig. 3C), the α5 and α6 regions of JD are in close contact with, and spread on the substrate, which results in complete α5-helix unfolding. The hairpin displays fluctuations (Fig. S7C and S8), moving from α-helix to random coil structure (from 0 to 450 ns, Rg about 1.85 nm), then from random coil to an antiparallel β-sheet solvent-exposed motif (from 450 to 700 ns, Rg decreased to 1.62 nm). This result is confirmed by the color maps of the secondary structure evolution during time (Fig. S9). Rg values of JD in its native conformation obtained from MD simulations are in good agreement with the Rg value estimated by SEC-FPLC (1.89 nm) (Fig. S1A and S1B). We actually assessed a Rg = 1.74 nm for JD in solvated environment at the equilibrium, and a Rg = 1.85 nm in the initial part of the JD-substrate simulations, when JD is still far from the surface.

**Figure 4:**
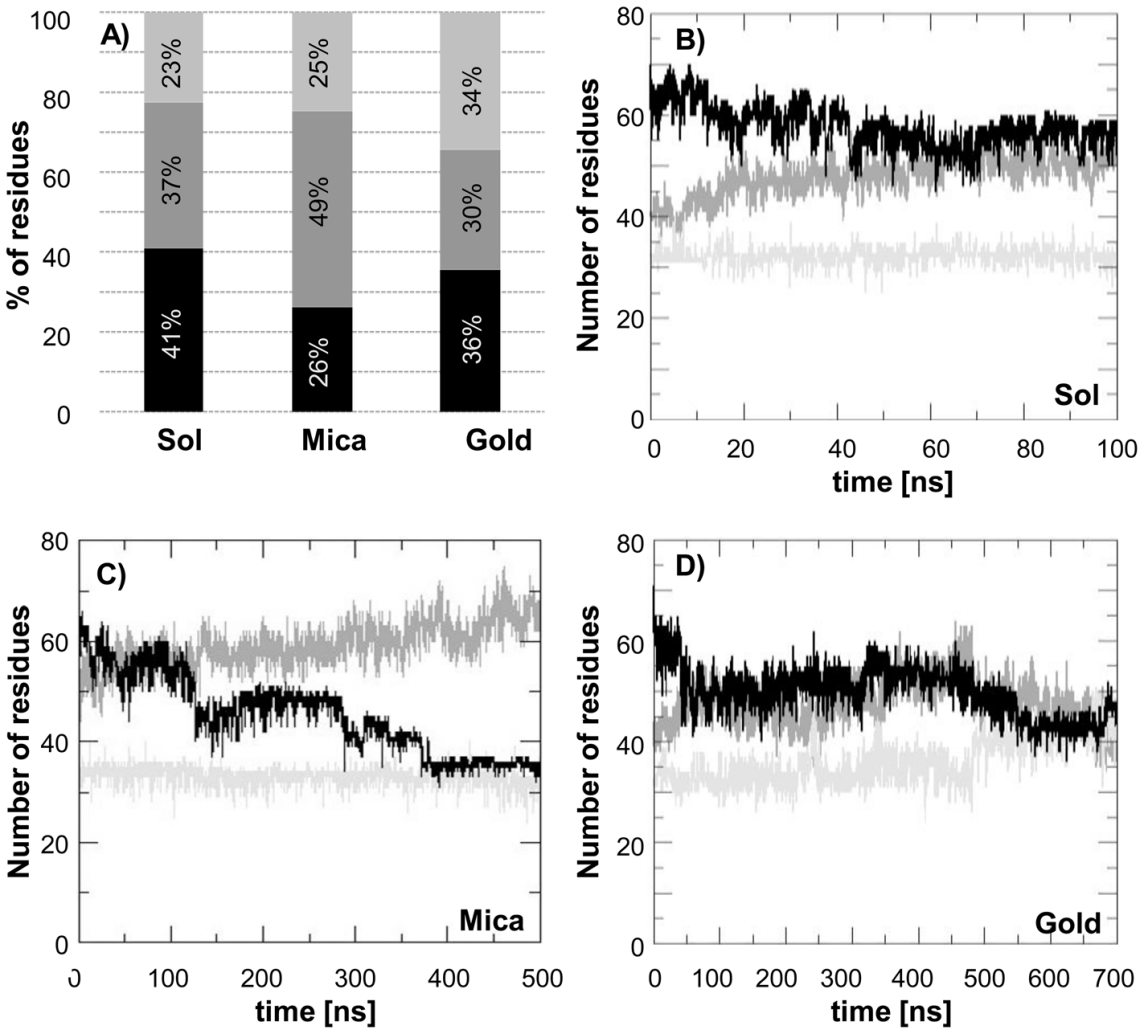
Percentage of residues organized in α-helix (black), coil (medium grey) and β-sheet (light grey) secondary structures. When JD is in contact with gold shows the highest value of β-sheet content with respect to JD in solution and in contact with mica (A). The number of residues arranged in α-helices (black), coils (medium grey) and β-sheets (light grey) as a function of time is reported for the JD in solution (B) on mica (C) and on gold (D).

Comparing the three different conditions here explored, the secondary structures of the protein change depending on the environment they are in contact with (Fig. 4). Figure 4A shows the percentage of residues arranged in a specific secondary structure, which makes it apparent that JD experiments a different conformation with a higher β-sheet content (34%) when is in contact with gold, compared to the two other cases (23% for JD in solution, and 25% for JD-mica system). Indeed, JD in solution (Fig. 4B) and JD-mica systems (Fig. 4C) show a similar behavior, featured by a constant number of residues arranged in β-sheets during time. In the presence of a hydrophilic surface, the coil structures markedly increase and the α-helices decrease, which points to an α-helix to coil transition. The same transition is also observed in the JD-gold system from 0 to 500 ns (Fig. 4D), but the extent of this transition is lower. A later important change in the JD secondary structure occurs after 500 ns, when the number of residues arranged in coils diminishes in favor of β-sheet formation, giving rise to a coil to β-sheet transition.

## Discussion

In the present study we investigated the interaction of the amyloidogenic protein domain JD with some solid surfaces. A largely hydrophobic and a hydrophilic substrate were assayed, i.e., gold and mica, respectively. Using this model system, we mainly aimed at providing insight into whether and how the interaction of amyloidogenic proteins with hydrophobic or hydrophilic molecular components and assemblies in the physiological milieu could affect protein stability and aggregation.

JD has a globular structure mainly consisting of β-sheets [27] and is endowed with an intrinsic tendency to form amyloid aggregates [13], both upon thermal stress [16], [31] and long incubation times at 37°C [17], [32]. So, despite its well-structured conformation it may undergo misfolding and aggregation, which implies that its adsorption to a surface might also result in structural changes. To shed light on this issue, we first monitored the process by AFM. However, AFM imaging alone can hardly provide hints as to the conformational state of a globular structure, although it can well reveal the appearance of aggregates. Thus, we also studied the conformational changes by FTIR and MD simulations.

In the case of JD deposition on mica, we observed conformational changes at t_0_, as proven by FTIR and MD simulations, but no aggregation. Thus, it seems that the interaction with the hydrophilic substrate is not strong enough to drive the aggregation [33], despite the intramolecular rearrangements. In contrast, when deposited on gold at t_0_, JD formed filaments, as imaged by AFM. This fits well with previous studies reporting that surface hydrophobicity can trigger protein aggregation through adsorption [34], [35]. In keeping with these observations, MD simulations showed that, although JD exposes the same binding site when interacting with either mica or gold, only the hydrophobic substrate is capable to mediate the aggregation. In this respect, it should be stressed that, despite the involvement of the same residues in the interaction with the substrate, namely Arg101 and Arg103, the mode is substantially different, interactions being effected in the former case via the charged guanidino groups, in the latter via the apolar aliphatic chains. This has major consequences in terms of protein conformational changes. In fact, mica does not favor adsorption, whereas JD spreads on gold, thus unmasking hydrophobic patches, whereby hydrophobicity provides an additional driving force for adsorption. This mechanism does not imply any energy cost [6] and gives rise to the formation of an initial adsorbed layer of JD molecules, which triggers aggregation and maintains the aggregation-prone binding sites for other molecules.

The α-helix to coil transition observed in the first time lapse of MD simulations and FTIR data evidence the key role of hydrophobic properties in the aggregation propensity of JD at t_0_. The coil to β-sheet transition observed in the second time lapse of MD simulation reveals a further structural change in favor of aggregation, which was not observed in FTIR experiments, where only intramolecular interactions could be detected at t_0_. This apparent contrast could be explained considering that our simulations model a single JD molecule interacting with a surface, thus reproducing the pure effect of surface properties on JD, which speed up the α-helix to coil and coil to β-sheet transitions. In contrast, FTIR experiments also detect the effect of intermolecular interactions, including the formation of a first adsorbed layer of JD molecules associated with native-structure to coil transition. This layer could be then responsible for protein-protein interaction, which does not favor a rapid coil to β-sheet transition as in the ideal case described by MD simulations.

The nature of the filamentous JD aggregates at t_0_ on gold also deserves some comments. At first glance, they might be regarded as amyloids. However, FTIR spectra clearly prove that they do not display any intermolecular β-sheet, one of the typical hallmarks of amyloids at any stage of aggregation.

Another remarkable finding of the present work is that at t_48_ the JD fails to assemble into filamentous aggregates, even in the presence of the gold substrate. Indeed, at t_48_ AFM detected globular structures and FTIR spectra revealed intermolecular β-sheets both in solution and in the presence of both substrates, with marginal differences between mica- and gold-exposed JD. On the whole, this pattern is characteristic of amyloid-like, oligomeric structures. Thus, although protein aggregates interact with the surface, no major modification of these assemblies is triggered by the adsorption. One can rationalize these findings by assuming that JD amyloid aggregation involves intermolecular hydrophobic interactions stronger than those with the substrate, whereby even gold is unable to displace them once they are formed. This may explain both the accumulation of large aggregates on gold only at t_0_, and the marginal effects of the hydrophilic mica substrate on the aggregation process. No less important, a completely different pattern of interaction with the substrates was observed for the non-amyloidogenic GST. The protein misfolded on gold under any adopted condition. These observations suggest that the gold substrate is capable of disrupting intramolecular hydrophobic interactions of native-like structures only.

The capability of hydrophobic surfaces to preferentially interact with amyloidogenic proteins, like in the case of the JD, is also relevant to the their mechanisms of toxicity. Actually, growing evidence shows that such mechanisms are mediated by interactions with the plasmamembrane, as a result of a complex interplay between the structural and physicochemical features of both the oligomers and the membrane itself [36]. Thus, our work suggests that the aggregation state of these proteins is of paramount importance in modulating the interactions with a hydrophobic surface. More specifically, our results make it apparent that beyond a certain aggregation stage such interactions cannot occur any more. In turn, they also can affect the mode of protein aggregation, which suggests that the physiological milieu can substantially modify the aggregation pathway.

## Materials and Methods

### Protein purification

JD was produced as previously described [17]. Glutathione S-tranferase (GST) was purified according to GE Healthcare Life Sciences-GST fusion protein Handbook (GE Healthcare Life Sciences, Little Chalfont, England). Proteins were stored in storage buffer (50 mM Tris-HCl, pH 8.0, 10 mM glutathione) at −20°C. Before use, the proteins were centrifuged at 15000× g for 15 min at 4°C to eliminate aggregates. Storage buffer was replaced by PBS (25 mM potassium phosphate, pH 7.2, 0.15 M NaCl), immediately prior to each experiment, using PD10 desalting columns (GE Healthcare LifeSciences, Little Chalfont, England). Protein content was determined using Comassie brilliant blue G-250 from Pierce (Thermo Scientific Rockford, IL USA) and bovine serum albumin as a standard protein.

### Substrate preparation

Mica and gold (80× 80 mm) were used as substrates for protein deposition. They share a very low (subnanometric) roughness. Mica is a phyllosilicate mineral of aluminium and potassium consisting of several flat sheets. It is chemically inert, atomically flat and easy to cleave by tweezers or adhesive tape. It is negatively charged in aqueous medium and hydrophilic. Gold substrates were produced by thermal evaporation of pure gold on mica in vacuum in an Edwards E306A evaporator, with the mica substrate heated at 400°C. The 50 nm thick metal film was then glued onto a silicon wafer by epoxy resin (at 140°C for 1 h) and subjected to template stripping [37], which revealed a very flat and clean surface (roughness 0.2–0.5 nm as measured by AFM).

### Protein preparation and analysis

25 µM JD or GST preparations were incubated in PBS at 37°C for 48 h. Before and after incubation, 100-µl samples were centrifuged at 14000× g for 15 min. The pellet was resuspended in 60 µl of Milly-Q water. 2-µl drops were deposited on substrates, washed with 50 µl of water and dried in air. AFM images were acquired in air in tapping mode by an Autoprobe CP Research (Thermomicroscope, Bruker) using a large area scanner (100× 100 µm maximum) and tips with 10 nm curvature radius. FTIR spectroscopy measurements (Materials and Methods S1: FTIR) on JD and GST were performed both in solution and on mica or gold substrates, where 2 µl of protein solution were deposited, rinsed with 50 µl of Milly-Q water and dried in air at room temperature. 6 mg/ml protein in PBS was analyzed immediately after purification and after 48 h of incubation at 37°C.

### MD simulations

Three molecular systems were modeled: the JD protein in solution, the JD-mica and the JD-gold systems, whereby the protein interacts with the respective substrates. JD molecular structure was obtained from the Protein Data Bank (PDB-code: 1YZB). The 53a6 GROMOS force-field (53a6) [38] was used for defining protein topology, while *ad hoc* topologies (Materials and Methods S1: MD. Models and Force-fields) were specified for mica [39] and gold [30]. Muscovite mica substrate was modeled as one single layer, while gold was modeled as three-layers gold substrate considering the polarization aspect of its metallic nature, due to the charge density of the adsorbed molecules [40]. MD simulations were carried out using GROMACS 4.5 [41]. Preliminary MD simulations were run to identify the most favorable interaction orientation of the protein faced to each specific substrate; six different initial orientations were tested for each molecular system and the preferential interaction sites on the protein outer surface were identified. For each molecular system in its favorable orientation, equilibrium MD simulations in explicitly solvated environment were performed at 300 K in the NVT ensemble to analyze in detail the conformational changes of the protein (Materials and Methods S1: MD. Simulations).

## Supporting Information

Figure S1 **Conformational analysis of freshly purified JD.** (A) SEC-FPLC profile of JD on a Superose 12 10/300 GL (GE Healthcare) in PBS buffer. (B) Kd (distribution coefficient) value of JD (red square) plotted against a reference set of standard proteins with known Rg values (gray squares). Coordinates of reference proteins were fitted to a linear equation (y = −1.2666x +0.7066) and Rg values for JD calculated. (C) Far-UV CD spectra of freshly purified JD at a 7 µM concentration.
(TIFF)

Figure S2 **SEM images of JD filaments on gold substrate at t0.**
(TIFF)

Figure S3 **Water contact angle on mica (A) and gold (B) surfaces.** Panel (C) shows a SEM image of drop solution of JD after drying on gold surface.
(TIFF)

Figure S4 **Aggregation kinetics of JD in solution monitored by FTIR spectroscopy and ThT fluorescence.** (A) The second-derivative ATR/FTIR spectra in the amide I band of JD in solution are reported at different incubation times at 37°C. The second-derivative spectrum of the insoluble aggregates, obtained by sample centrifugation after 72 h of incubation (72 h pellet), is also given. The arrows point to increasing time of incubation. Spectra are presented after normalization at the ∼1515 cm-1 Tyr band. The band assignment of the main peaks to the protein secondary structures are indicated. (B) ThT fluorescence of protein incubated at 37°C at a 6 mg/ml concentration in PBS, pH 7.2, and in the presence of 20 mM ThT. Fluorescence was recorded using a plate reader, with values read every 30 min. Individual values are the mean of three independent determinations, with standard deviations never exceeding 5% of the mean.
(TIFF)

Figure S5 **Mass spectrometry analysis of JD.** ESI-MS spectra (A, C) and mass deconvolution (B, D) of JD before (A, B) and after (C, D) incubation. 19 µM protein in 5 mM ammonium acetate, 1% formic acid (A) or 7.5 µM in 2.5 mM ammonium acetate, 1% formic acid, 50% acetonitrile (C). The most intense peaks in panels A and C are labeled with the corresponding charge state of the protein.
(TIFF)

Figure S6 **Contact surface (A, C) and minimum distance of the residues (B, D) of JD interacting with gold (A, B) and mica surface (C, D).** The black curve is related to MD simulations of JD with Arg residues in position 101 and 103, while the red curves are related to MD Ala scan simulations, where the two Arg residues were replaced by Ala residues.
(TIFF)

Figure S7 **Root Mean Square (RMS) fluctuations of JD protein.** (A) in solution averaged in the 0–10 ns range (black) and in the 90–100 ns range (red), where the α2–α3 hairpin residues (light grey region in the RMS fluctuation graphs) show large fluctuations; (B) in contact with mica surface averaged in the 0–10 ns range (black) and in the 490–500 ns range (red), where hairpin fluctuations decay; (C) in contact with gold surface averaged in the 0–10 ns range (black), in the 390–400 ns range (blue), where the hairpin fluctuations are still present, and in the 690–700 ns range, where hairpin fluctuations decay (red). Looking at the binding site of the protein with the surface (residues Arg101 and Arg103, dark grey region in the RMS fluctuation graphs), it can be noticed that fluctuations, which are present for the protein in solution and in the initial phase of the protein-surface interaction, strongly decay when the site is in contact with the surface (red curve in B and red and blue curves in C).
(TIFF)

Figure S8 **Root Mean Square Deviation (RMSD) of JD protein faced to gold surface (A) where two different stable regions are detectable, the first one from 25 to 450 ns and the second one from 500 to 700 ns.** The first stable region is characterized by a hairpin domain fluctuating far from the globular core of the protein; the secondary structure of the hairpin experiments an α-helix to coil transition (see panel B representing the protein structure at 50 ns, where the hairpin domain is still arranged in a double helix structure and panel C, which shows the protein conformation at 400 ns with the hairpin domain formed by coil structures). The second stable region is characterized by a coil to β-sheet transition, as shown in panel C at 400 ns and panel D at 600 ns, where the hairpin domain is structured in an antiparallel β-sheet. Thus, the hairpin does not fluctuate anymore and it is in contact with the gold surface.
(TIFF)

Figure S9 **Detail of the time-dependent secondary structures evolution of the α2–α3 hairpin region (residues from 31 to 62) for the protein in solution (A), in contact with mica (B) and gold (C) surfaces.** The presence of mica causes a change in the secondary structure from α-helices (blue) to coils (white), while the gold surface induces a two-step transition: the first from α-helices to coils and the second (at about 450 ns) from coils to β-sheets (red). The β-sheet structure formed in contact with gold surface is stable until 700 ns of MD simulation.
(TIFF)

Materials and Methods S1 **Supplemental materials and methods.**
(DOC)
