# Supplementary figures and images for: A Hydrophobic Gold Surface Triggers Misfolding and Aggregation of the Amyloidogenic Josephin Domain in Monomeric Form, While Leaving the Oligomers Unaffected
Source: PLoS One. 2013 Mar 19;8(3):e58794. doi: 10.1371/journal.pone.0058794 (PMC3602447; doi:10.1371/journal.pone.0058794)

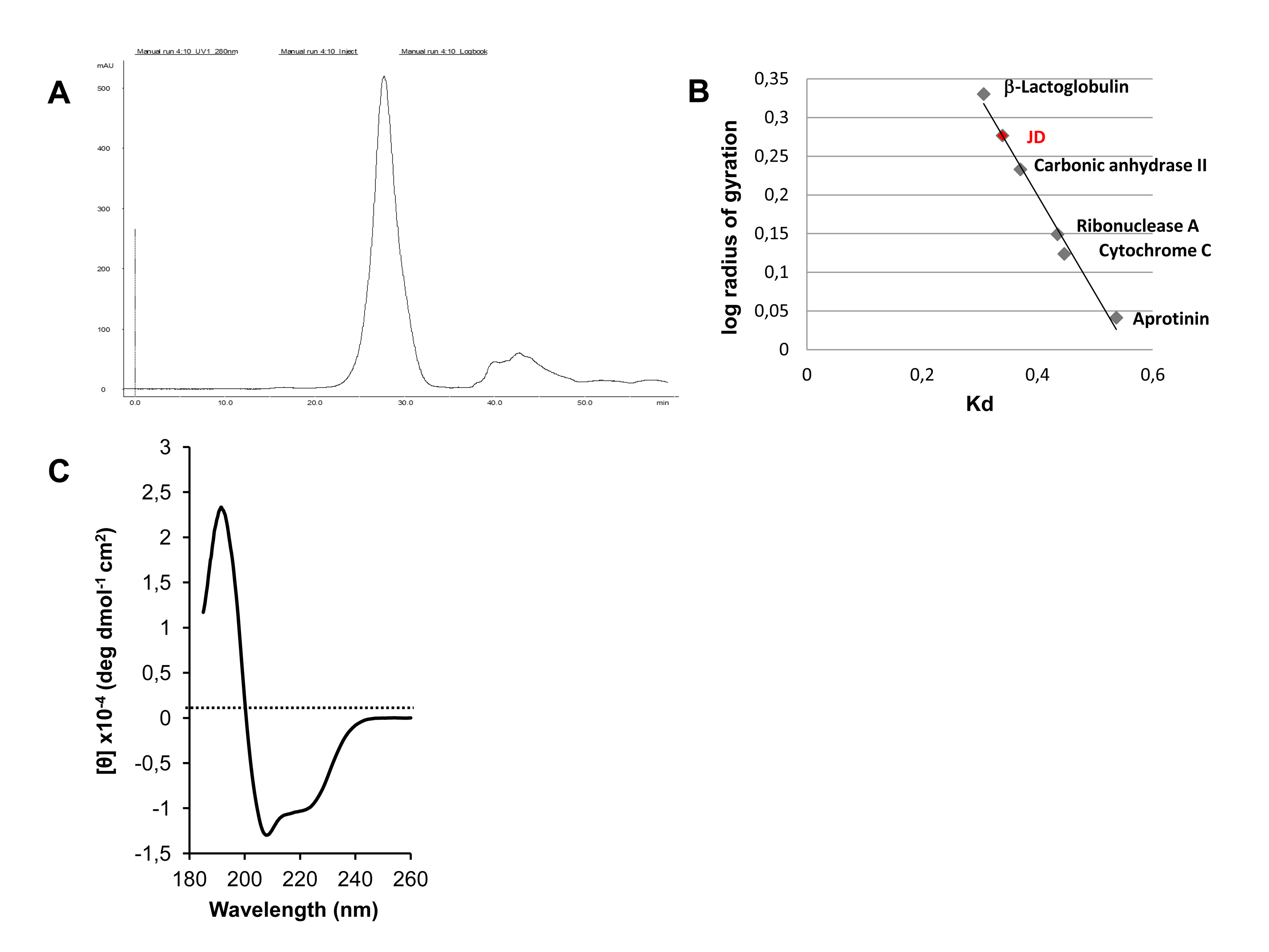

Supplement: Figure S1 — Conformational analysis of freshly purified JD. (A) SEC-FPLC profile of JD on a Superose 12 10/300 GL (GE Healthcare) in PBS buffer. (B) Kd (distribution coefficient) value of JD (red square) plotted against a reference set of standard proteins with known Rg values (gray squares). Coordinates of reference proteins were fitted to a linear equation (y = −1.2666x +0.7066) and Rg values for JD calculated. (C) Far-UV CD spectra of freshly purified JD at a 7 µM concentration. (TIFF) [file pone.0058794.s001.tiff]

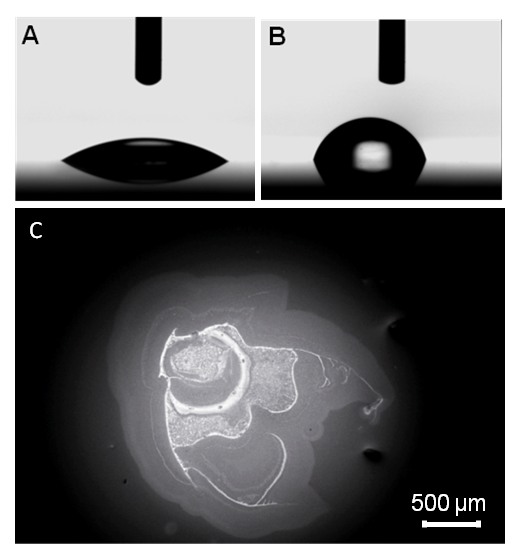

Supplement: Figure S3 — Water contact angle on mica (A) and gold (B) surfaces. Panel (C) shows a SEM image of drop solution of JD after drying on gold surface. (TIFF) [file pone.0058794.s003.tiff]

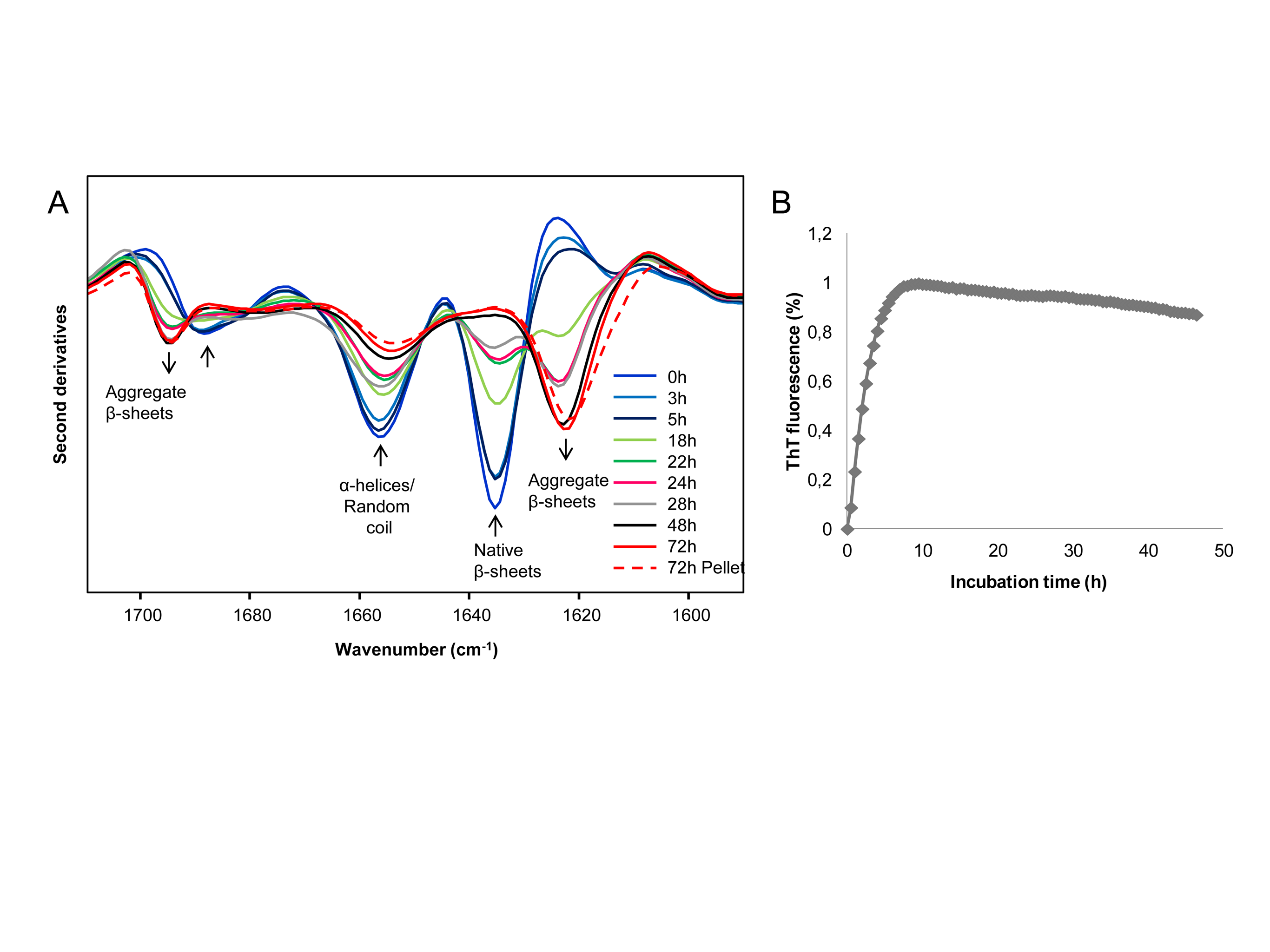

Supplement: Figure S4 — Aggregation kinetics of JD in solution monitored by FTIR spectroscopy and ThT fluorescence. (A) The second-derivative ATR/FTIR spectra in the amide I band of JD in solution are reported at different incubation times at 37°C. The second-derivative spectrum of the insoluble aggregates, obtained by sample centrifugation after 72 h of incubation (72 h pellet), is also given. The arrows point to increasing time of incubation. Spectra are presented after normalization at the ∼1515 cm-1 Tyr band. The band assignment of the main peaks to the protein secondary structures are indicated. (B) ThT fluorescence of protein incubated at 37°C at a 6 mg/ml concentration in PBS, pH 7.2, and in the presence of 20 mM ThT. Fluorescence was recorded using a plate reader, with values read every 30 min. Individual values are the mean of three independent determinations, with standard deviations never exceeding 5% of the mean. (TIFF) [file pone.0058794.s004.tiff]

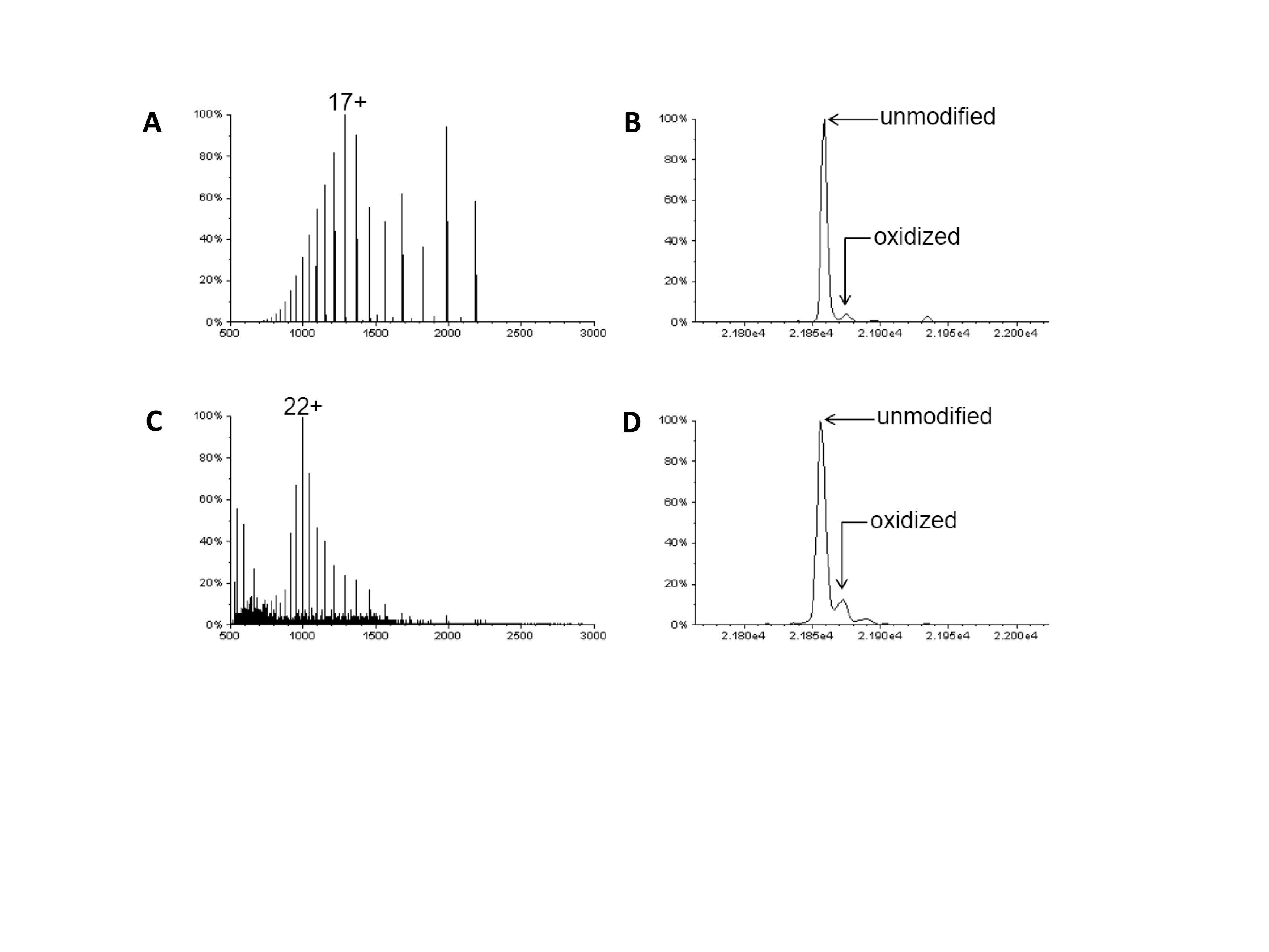

Supplement: Figure S5 — Mass spectrometry analysis of JD. ESI-MS spectra (A, C) and mass deconvolution (B, D) of JD before (A, B) and after (C, D) incubation. 19 µM protein in 5 mM ammonium acetate, 1% formic acid (A) or 7.5 µM in 2.5 mM ammonium acetate, 1% formic acid, 50% acetonitrile (C). The most intense peaks in panels A and C are labeled with the corresponding charge state of the protein. (TIFF) [file pone.0058794.s005.tiff]

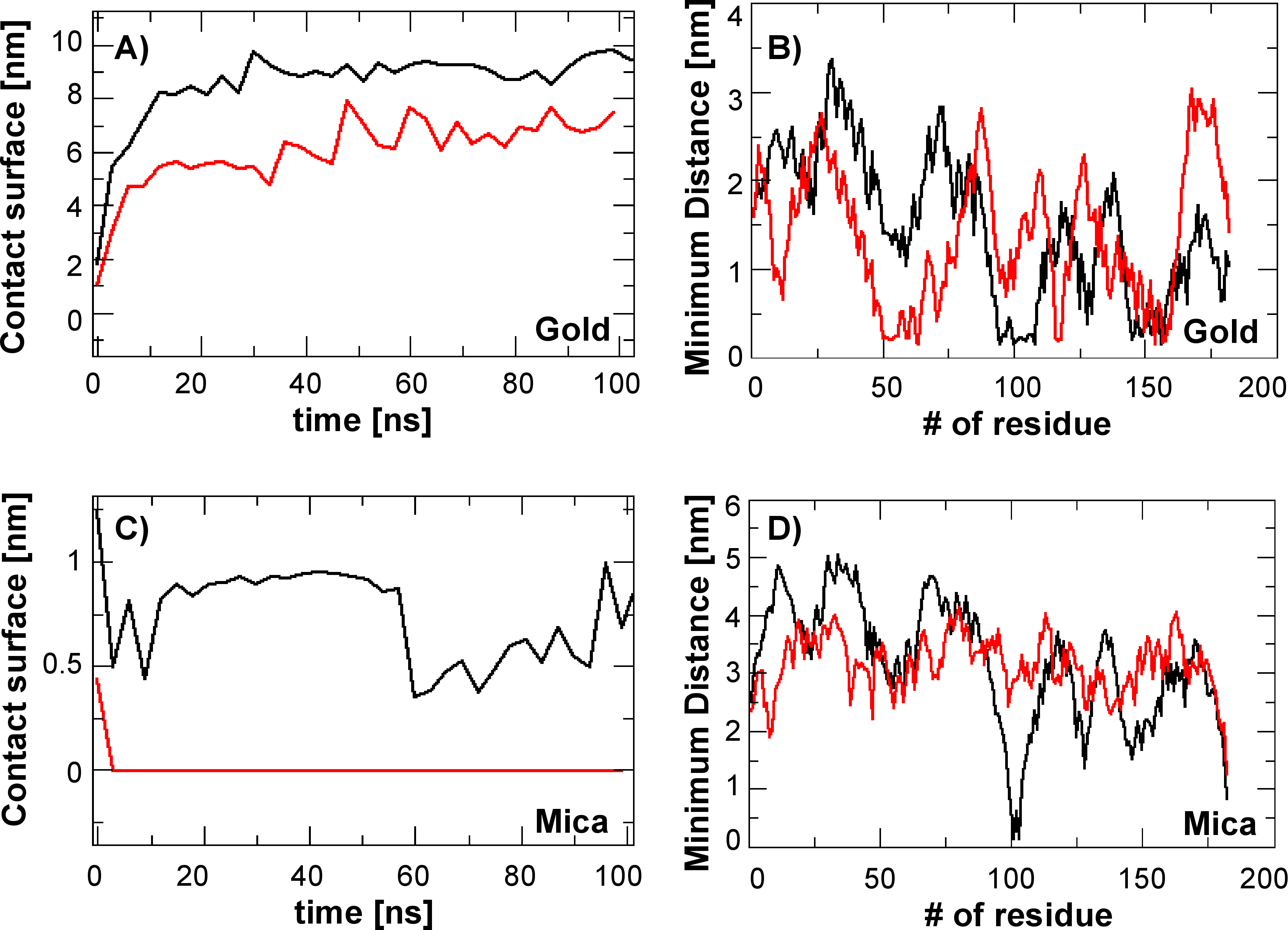

Supplement: Figure S6 — Contact surface (A, C) and minimum distance of the residues (B, D) of JD interacting with gold (A, B) and mica surface (C, D). The black curve is related to MD simulations of JD with Arg residues in position 101 and 103, while the red curves are related to MD Ala scan simulations, where the two Arg residues were replaced by Ala residues. (TIFF) [file pone.0058794.s006.tiff]

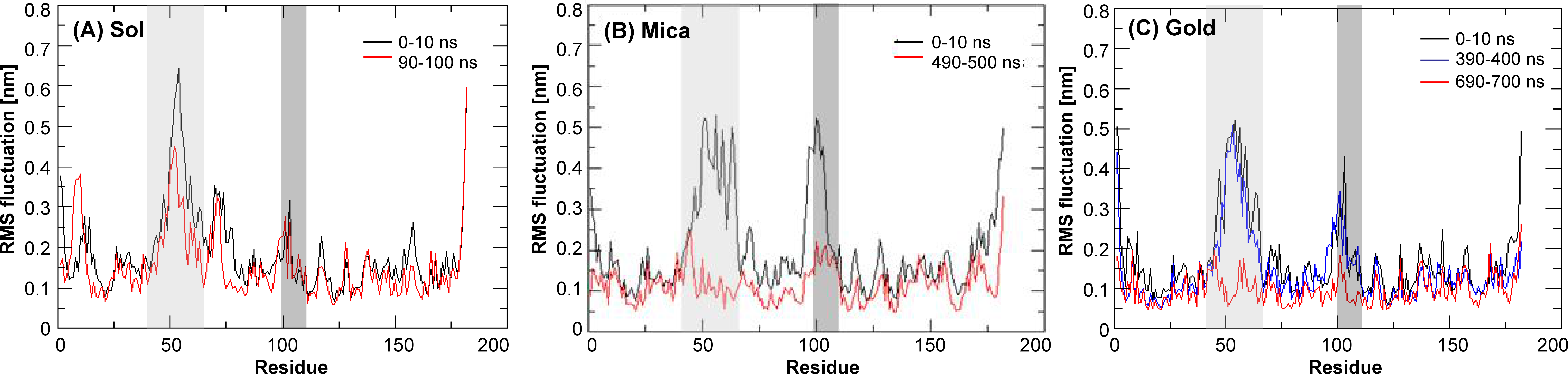

Supplement: Figure S7 — Root Mean Square (RMS) fluctuations of JD protein. (A) in solution averaged in the 0–10 ns range (black) and in the 90–100 ns range (red), where the α2–α3 hairpin residues (light grey region in the RMS fluctuation graphs) show large fluctuations; (B) in contact with mica surface averaged in the 0–10 ns range (black) and in the 490–500 ns range (red), where hairpin fluctuations decay; (C) in contact with gold surface averaged in the 0–10 ns range (black), in the 390–400 ns range (blue), where the hairpin fluctuations are still present, and in the 690–700 ns range, where hairpin fluctuations decay (red). Looking at the binding site of the protein with the surface (residues Arg101 and Arg103, dark grey region in the RMS fluctuation graphs), it can be noticed that fluctuations, which are present for the protein in solution and in the initial phase of the protein-surface interaction, strongly decay when the site is in contact with the surface (red curve in B and red and blue curves in C). (TIFF) [file pone.0058794.s007.tiff]

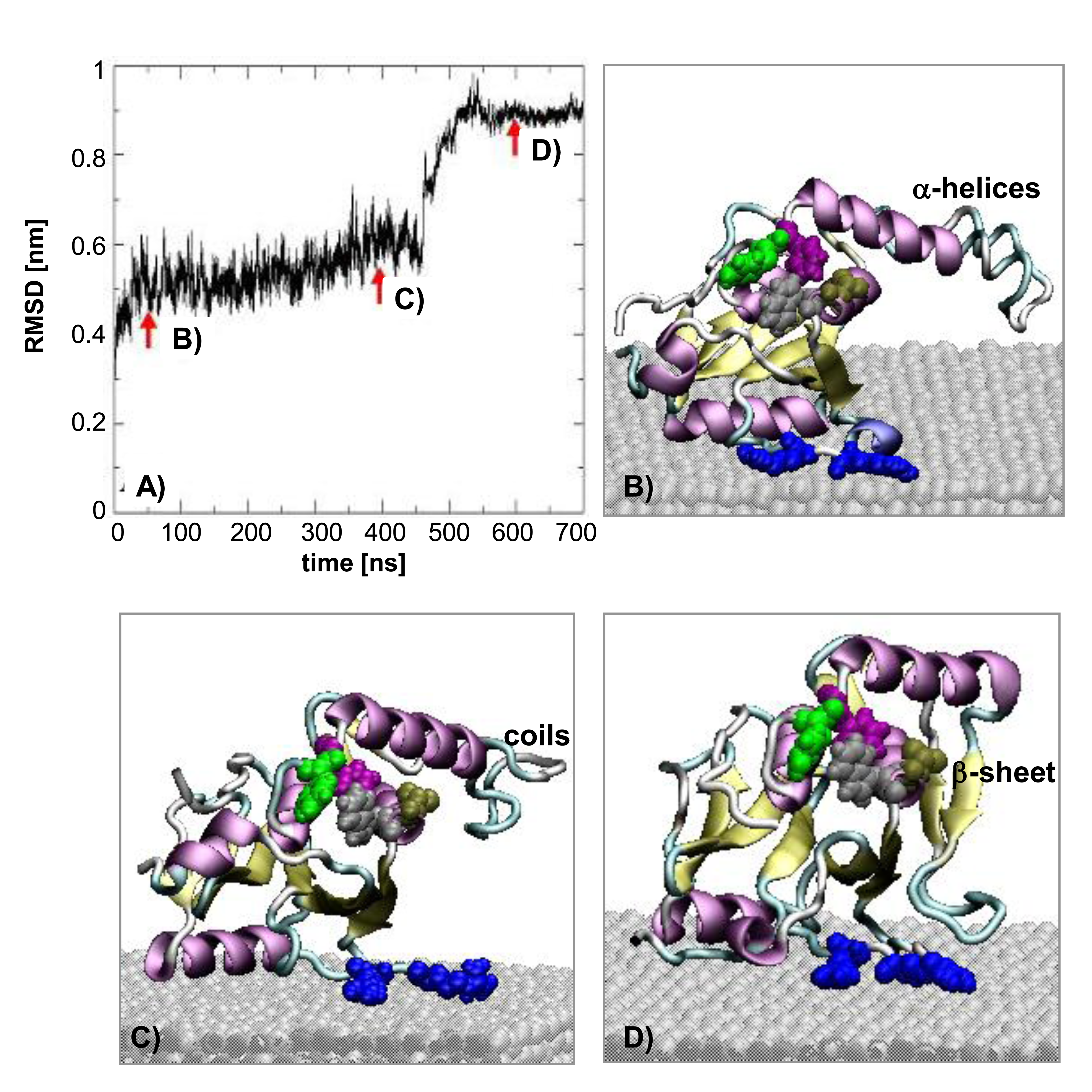

Supplement: Figure S8 — Root Mean Square Deviation (RMSD) of JD protein faced to gold surface (A) where two different stable regions are detectable, the first one from 25 to 450 ns and the second one from 500 to 700 ns. The first stable region is characterized by a hairpin domain fluctuating far from the globular core of the protein; the secondary structure of the hairpin experiments an α-helix to coil transition (see panel B representing the protein structure at 50 ns, where the hairpin domain is still arranged in a double helix structure and panel C, which shows the protein conformation at 400 ns with the hairpin domain formed by coil structures). The second stable region is characterized by a coil to β-sheet transition, as shown in panel C at 400 ns and panel D at 600 ns, where the hairpin domain is structured in an antiparallel β-sheet. Thus, the hairpin does not fluctuate anymore and it is in contact with the gold surface. (TIFF) [file pone.0058794.s008.tiff]

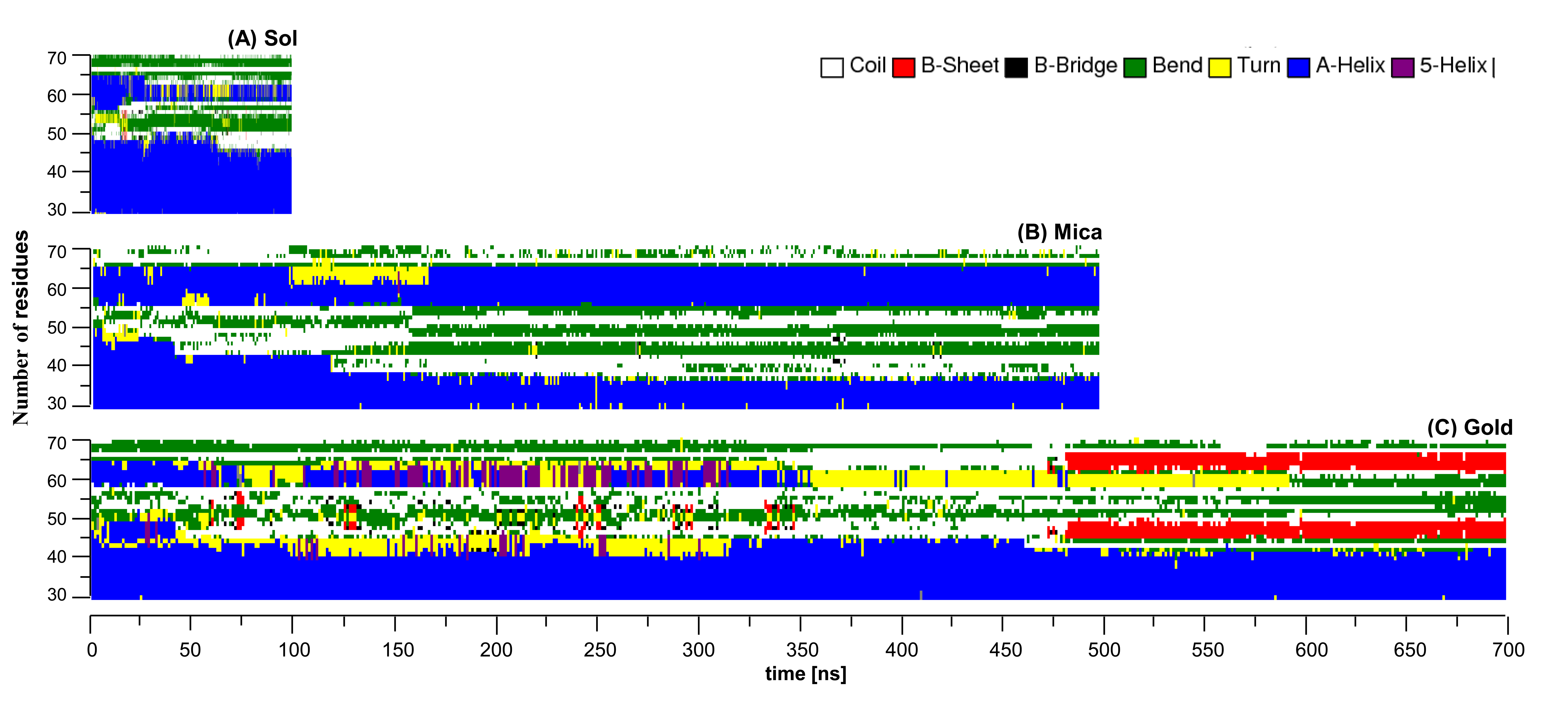

Supplement: Figure S9 — Detail of the time-dependent secondary structures evolution of the α2–α3 hairpin region (residues from 31 to 62) for the protein in solution (A), in contact with mica (B) and gold (C) surfaces. The presence of mica causes a change in the secondary structure from α-helices (blue) to coils (white), while the gold surface induces a two-step transition: the first from α-helices to coils and the second (at about 450 ns) from coils to β-sheets (red). The β-sheet structure formed in contact with gold surface is stable until 700 ns of MD simulation. (TIFF) [file pone.0058794.s009.tiff]
