# Supplementary material for: A Hydrophobic Gold Surface Triggers Misfolding and Aggregation of the Amyloidogenic Josephin Domain in Monomeric Form, While Leaving the Oligomers Unaffected
Source: PLoS One. 2013 Mar 19;8(3):e58794. doi: 10.1371/journal.pone.0058794 (PMC3602447; doi:10.1371/journal.pone.0058794)
Supplement: Materials and Methods S1 — Supplemental materials and methods. (DOC) [file pone.0058794.s010.doc]

**Supporting information Apicella et al.**

**MATERIALS AND METHODS**

**Imaging.**

SEM observations were performed on the same samples without any conducting coating. This was accomplished using low accelerating voltage (3 kV) in order to avoid charging effects and electron damage. Fig. S1 shows the presence of bundle structures of JD on gold at t_0_. Fiber lateral dimensions, as measured by SEM, are in agreement with those measured by AFM. SEM analysis shows the presence of complex bundles, which appear in AFM images as large protrusions.

**Surface wettability.**

To characterize mica and gold substrates wettability, static water contact angle measurements were performed using an optical video contact angle system (OCA-15-plus, Dataphysics). A 2-μl droplet of water (Chromasolv water for HPLC, Sigma Aldrich), having a surface tension of 78.2 mN/m, was dispensed on the samples using the electronic syringe unit of the instrument equipped with a 500-μl Hamilton syringe. The static contact angle was measured using the sessile drop method with dedicated software (SCA 2.0) determining the contact angle based on the Young-Laplace fitting. Results reported in Fig. S2 indicate a mean value of the contact angle of 33° on mica and 75° on gold.

**FTIR.**

FTIR spectra of proteins in solution and on mica and gold surfaces were collected in attenuated total reflection (ATR) mode on a single reflection diamond plate (Golden Gate, Specac, USA) using the Varian 670-IR spectrometer (Varian Australia Pty Ltd, Mulgrave VIC, Australia) equipped with a nitrogen cooled Mercury Cadmium Telluride (MCT) detector under the following conditions: 2 cm^-1^ resolution, a scan speed of 25 kHz, 1000 scans co-addition and triangular apodization. For the protein in solution, an aliquot of 5 μl was deposited on the ATR plate and measured as protein film after water evaporation [1]. In the case of mica and gold substrates, 2-μl aliquots of the protein solution were deposited on the substrates, rinsed with 50 μl of Milly-Q water and dried in air at room temperature. The substrates were then placed in contact with the ATR element to collect the FTIR spectra of the adsorbed protein. Measured spectra were smoothed by a binomial function (11 points) and second derivative spectra were obtained by the Savitsky–Golay method (3rd grade polynomial, five smoothing points) using the Grams/AI software (Thermogalactic, USA).

**MD. Models and Force-fields.**

Details of all the molecular models employed to investigate the behavior of JD and its interactions with hydrophilic (mica) and hydrophobic (gold) substrates are reported below.

*JD:* the atomic structure used to model JD protein was taken from Protein Data Bank (PDB-code: 1YZB). The atomistic model consisting of 182 residues was solved using NMR techniques [2]. The 53a6 GROMOS force-field (53a6) [3] was used for defining the protein topology.

*Mica:* Muscovite mica (K Al_2_ (AlS3O_10_)(OH)_2_) is a stack of 10-Å-thick sheets. Each sheet consists of two oppositely-oriented layers of tetrahedrons that are linked together by coordinated cations in octahedral fashion. The molecular model of mica surface built for MD simulations consists of one sheet of mica cleaved along the (001) plane. Force-field parameters for mica-type silicates were set in Gromacs code according to literature data [4].

*Gold***:** The film deposition of gold on mica surface, which is the method used for the preparation of the substrates for experimental investigation, is a process whereby crystal gold orientations cannot be controlled. Then, a (111) gold surface model was used for MD simulations, since i) it is considered to be stable in contact with air and water, ii) it has a reduced reactivity and iii) it was tested in several experimental studies where small molecules were adsorbed to the surface with the aim of setting the force-field parameters and validating the force-field quality. A force-field specifically developed for biological molecules was implemented in GROMACS code [5]; the model of gold substrate consisted of a three-layers gold substrate where the polarization aspect of metallic nature of gold due to the charge density of the adsorbed molecules was taken into account by means of the image interaction method.

**MD. Simulations.**

Three different molecular systems were modeled and MD simulations were run; i) the JD molecular system, consisting of the protein alone in physiologic water-like environment, ii) the JD-mica system, where JD is faced to the mica substrate modeling the hydrophilic surface, and iii) the JD-gold system, where the protein is positioned close to a hydrophobic gold substrate. All the molecular systems are simulated in an explicit water-like environment. MD simulations were carried out using GROMACS 4.5 [6]. MD simulation of JD alone in water environment provides information on the JD behavior to be used as reference conformation of the protein. The simulations of the protein-surface systems followed a double step procedure. First, for each protein-substrate molecular system, six preliminary MD simulations were carried out in order to identify preferential specific interaction sites of JD protein for the specific substrate (gold or mica). Each simulation was characterized by a different initial orientation of the protein with respect to the surface obtained by rigidly rotating the protein of about 90° around one of the three Cartesian axes while maintaining the substrate fixed. Then, once the preferential interaction surface was identified, for each protein-substrate system in water (JD-mica system and JD-gold system) a long-lasting MD simulation was carried out. Details related to the above mentioned preliminary MD simulations are reported in the following, together with information related to long-lasting simulations performed for data analysis.

*Preliminary simulations.* The JD molecule was surrounded by explicitly modeled water molecules (Simple Point Charge water model) in a box of 12 X 9 X 9 nm of size. The two other systems (JD-mica and JD-gold molecular systems) in six different initial orientations were simulated in explicit water and inserted in a box with size of about 12 x 11 x 11 nm. Each molecular system was energy minimized for 1000 steps by using the steepest descent algorithm. A first position restraining MD simulation was carried out for 50 ps in the NTV canonical ensemble at 300 K with an integrating time step of 2 fs. Temperature was controlled using a weak couple scheme. Electrostatic interactions were treated with a Particle Mesh Ewald (PME) approach, with a 12-Å grid and a fourth order spline interpolation. Lennard Jones interactions were cut off at a distance of 9 Å, with a smooth switch-off starting at a distance of 10 Å. To permit full electrostatic calculation via PME summation, the JD-substrate systems were made periodic in all directions.

*Running simulations:* After the preliminary position restraining, MD simulations were carried out for each system until equilibrium was reached. By associating a virtual site approach to the LINCS constraint solver (selecting all-bonds constraint), a time step of 4 fs was used. In a virtual site approach the bond-angle vibrations involving hydrogen atoms are removed and the masses are properly modified increasing the moment of inertia of the water molecules, the hydroxyl, the sulfhydryl, and the amine groups, without affecting the equilibrium properties of the system and its dynamical properties. In particular, the following simulations were run and used to analyze and compare data of the different molecular systems with the aim of identifying the conformational changes induced by different environmental conditions mediated by different substrate properties.

1. JD in water: MD simulation of JD in water was performed with an integration time step of 4 fs for a total time of 100 ns, where the last 50 ns were used for data analysis.
2. JD-Mica: MD simulation of the JD-Mica system was performed with an integration time step of 4 fs for a total time of 500 ns, where the last 100 ns were used for data analysis.
3. JD-Gold: MD simulation of the JD-Gold system was run with an integration time step of 4 fs for 700 ns, a time lapse long enough to achieve a stable conformation of the protein in contact with the surface. The last 100 ns were used for data analysis.

**References**

1. Natalello A, et al. (2011) A Major Role for Side-Chain Polyglutamine Hydrogen Bonding in Irreversible Ataxin-3 Aggregation. PLoS ONE 6(4): e18789.
2. Nicastro G, et al. (2005) Solution structure of the Josephin domain of ataxin-3. Proc Natl Acad Sci USA 102: 10493-10498.
3. Oostenbrink C, et al. (2005) Validation of the 53A6 GROMOS force field. Eur Biophys J 34: 273-284.
4. Heinz H, et al. (2005) Force field for mica-type silicates and dynamics of octadecylammonium chains grafted to montmorillonite. Chem Mater 17: 5658-5669.
5. Iori F, et al. (2009) GolP: an atomistic force-field to describe the interaction of proteins with Au(111) surfaces in water. J Comput Chem 30: 1465-1476.
6. Hess B, et al. (2008) GROMACS 4: Algorithms for Highly Efficient, Load-Balanced, and Scalable Molecular Simulation. J. Chem. Theory Comput 4(3): 435-447.
